# Relationship Between Nucleos(t)ide analogue antiviral response time and prognosis in Chronic Hepatitis B: conclusions depend on baseline viral load and HBeAg status

**DOI:** 10.3389/fphar.2025.1572827

**Published:** 2025-04-24

**Authors:** Jingtao Huang, Xiangyong Li, Lijie You, Yutian Chong, Hong Cao

**Affiliations:** Department of Infectious Diseases, Third Affiliated Hospital of Sun Yat-sen University, Sun Yat-sen University, Guangzhou, China

**Keywords:** antiviral agent, chronic hepatitis B, HBV, cumulative incidence, liver disease, viral load

## Abstract

**Introduction:**

Current guidelines for changing antiviral therapy regimens do not consider different baseline statuses. This study aimed to investigate whether significant prognostic differences exist among patients achieving virological response at various time points and whether these differences vary by viral load and HBeAg status.

**Methods:**

This retrospective cohort study included 1,037 patients, who were classified based on their baseline viral load (high viral load, HVL, ≥ 7log_10_ IU mL^−1^: 522 individuals; or non-HVL, < 7log_10_ IU mL^−1^: 515 individuals) and HBeAg status (positive: 668 individuals; or negative: 369 individuals). Based on the virological response time, patients were grouped separately using 48 weeks and 96 weeks as the boundaries. The prognoses across these groups were evaluated and compared.

**Results:**

Patients in the within-48-week group, 48–96-week group, and after-96-week group exhibited no significant difference in the incidence of liver disease progression (5.08% vs 4.38% vs 9.61%, *p* = 0.33). For HVL or HBeAg-positive patients, there was no significant difference in the cumulative incidence of liver disease progression between the within-48-week group and the 48–96-week group. In contrast, for non-HVL or HBeAg-negative patients, the cumulative incidence of liver disease progression was significantly higher in the after-48-week group than in the within-48-week group. Only the after-96-week group showed a significant increase in maintained virological response rate (*p* = 0.04).

**Conclusion:**

Antiviral treatment should be adjusted at 48 weeks for non-HVL or HBeAg-negative patients. For HVL and HBeAg-positive patients, we suggest changing the antiviral treatment plan to 96 weeks.

## 1 Introduction

Chronic hepatitis B (CHB) is a globally prevalent infectious disease caused by hepatitis B virus (HBV). Despite widespread HBV vaccination and advances in clinical diagnosis and treatment, which have significantly decreased the mortality rate, many patients remain affected. The World Health Organization reported that in 2022, over 1,100,000 people worldwide died from severe liver conditions associated with HBV infection, including cirrhosis, liver failure, and primary hepatic carcinoma (*Implementing the global health Secter strategies on HIV, viral hepatitis and sexually transmitted infections*, 2022–2030).

Antiviral therapy is the cornerstone of CHB treatment, and current options include interferons and nucleoside/nucleotide analogues (NAs). Owing to their lower efficacy and higher incidence of interferon adverse reactions, NAs are recommended as the primary treatment. Globally recommended first-line NAs for CHB treatment include entecavir (ETV), tenofovir disoproxil (TDF), tenofovir alafenamide (TAF), and tenofovir amibufenamide (TMF) (European Association for the Study of the Liver and EASL, 2017; Terrault et al., 2018; Lau et al., 2021; You et al., 2023). After 48 weeks of treatment with any of these drugs, the overall virological response (VR) rate in the general population ranges from 75% to 85% (Marcellin et al., 2008; Ceylan et al., 2013; Hou et al., 2015; Yan et al., 2015; Byrne et al., 2018; Kayaaslan et al., 2018; Liu et al., 2021), with significant variations across different patient groups. Over 90% of patients with HBeAg-negative status reach this milestone within 48 weeks of NA therapy, while the response rate for those with HBeAg-positive status is lower, at 60%–75% within the same timeframe (Marcellin et al., 2008; Hou et al., 2015; Byrne et al., 2018; Liu et al., 2021). Several studies have shown that only 50%–70% of patients with CHB and high viral load (HVL) at baseline achieve VR within 48 weeks (Fung et al., 2015; Yan et al., 2015). However, VR rates for all patient groups increase to approximately 90% when treatment is extended to 96 weeks (Fung et al., 2015; Ha et al., 2016; Kayaaslan et al., 2018). These two factors correlate: patients who are HBeAg-positive typically exhibit high viral loads, generally exceeding 20,000 IU/mL (Jeng et al., 2023). HBeAg serves as a marker of HBV DNA replication and infectivity in the natural history of HBV infection. The serological conversion of HBeAg implies that the replication of HBV DNA is trending towards cessation. Another study identified viral load and hepatitis B e-antigen (HBeAg) positivity at baseline as independent factors that delay VR (Li et al., 2019).

Studies have shown that non-VR and persistent low-level viremia are independent risk factors for cirrhosis and hepatocellular carcinoma (Jang et al., 2018; Nam et al., 2018; Zhang et al., 2021). Therefore, the latest Chinese Guidelines for the Prevention and Treatment of Chronic Hepatitis B recommend adjusting the treatment regimen for patients with detectable HBV DNA levels after 48 weeks of NA therapy, provided compliance or detection errors have been ruled out (You et al., 2023). This indicates that approximately 30% of patients with HVL at baseline or HBeAg-positive status undergoing antiviral therapy require treatment adjustment, which could affect treatment adherence, increase the risk of adverse reactions, and heighten the likelihood of developing resistance to multiple drugs (Lu et al., 2013; Liu et al., 2014). However, studies on whether notable disparities exist in medium-to long-term rates of maintained virological response (MVR) and the incidence rate of HBV-related liver disease progression between patients who achieve VR within 48 weeks and those who achieve VR within 48–96 weeks are limited. Whether these findings remain consistent across subgroups of patients with different baseline HBV DNA levels and HBeAg status is also unclear.

Therefore, this retrospective cohort study aimed to explore the correlation between the time to VR after NA treatment and the cumulative incidence of HBV-related liver disease progression. By clarifying this relationship, we aimed to determine the optimal timing for adjusting antiviral treatment regimens in various patient subgroups and provide evidence-based medical guidance for clinical practice.

## 2 Materials and methods

This study was a retrospective cohort analysis of data from 5,000 patients who received monotherapy with ETV or tenofovir (including TAF, TDF, and TMF) as antiviral treatment at the Third Affiliated Hospital of Sun Yat-sen University between 2000 and 2024. Patients were included based on the following criteria: (1) diagnosis of chronic viral hepatitis with an initial viral load of >100 IU mL^−1^, (2) undergoing antiviral therapy for the first time or resuming therapy after discontinuation for >2 years, (3) having a follow-up period of at least 96 weeks from antiviral treatment initiation, and (4) achieving virological response (VR) after antiviral therapy. The exclusion criteria were as follows: (1) the presence of other viral hepatitis infections, autoimmune liver diseases, or conditions significantly affecting liver function; (2) the presence of advanced liver disease (cirrhosis, liver cancer, or liver failure) at baseline; (3) missing critical data that significantly hindered the analysis; (4) discontinuation or alteration of antiviral treatment before achieving VR; and (5) noncompliance or adverse events with medication during follow-up. This study began with the initial administration of medication to patients who met the inclusion criteria and ended with the last review record before June 2024, liver transplantation, or patient death.

The Medical Ethics Committee of the Third Affiliated Hospital of Sun Yat-sen University approved this study protocol. Informed consent was obtained from all the participants included in this study (Approval Document Number II2025-077-11).

### 2.1 Study design

Patients who met the inclusion criteria were categorized into three groups based on how long they took to achieve VR: within-48-week group, 48–96-week group, and after-96-week group. The groups were compared to assess disparities in the cumulative incidence of HBV-related liver disease progression and the rate of achieving MVR during the study period. Subsequently, patients were further categorized according to their baseline viral load into the HVL (HBV DNA ≥ 7log_10_ IU mL^−1^) and non-HVL (HBV DNA < 7log_10_ IU mL^−1^) groups, and the comparisons were repeated. Comparisons were also made based on baseline HBeAg status. The objective was to determine whether including the baseline viral load and HBeAg status significantly influenced the outcomes.

### 2.2 Definitions

HVL is defined as a virological load of ≥7log_10_ IU mL^−1^ (European Association for the Study of the Liver and EASL, 2017), and the baseline refers to the time when patients began antiviral therapy. VR is defined as three consecutive viral load measurements <100IU/ml or below the current lower limit of detection after initiating antiviral therapy, with each test conducted at least 2 months apart. The time from baseline to achieving VR was recorded as the VR time. HBV-related liver disease progression events during cohort follow-up are defined as follows: (1) cirrhosis confirmed by imaging; (2) primary liver carcinoma, including hepatocellular carcinoma and intrahepatic cholangiocarcinoma (Zhou et al., 2010); and (3) meeting the clinical criteria for liver failure as outlined in the 2018 Guidelines for Diagnosis and Management of Liver Failure (Liver Failure and Artificial Liver Group, Chinese Society of Infectious Diseases, Chinese Medical Association and Severe Liver Disease and Artificial Liver Group, Chinese Society of Hepatology, Chinese Medical Association, 2019). MVR is defined as maintaining a virological load below 100 IU mL^−1^ in all tests conducted after achieving a VR.

### 2.3 Statistical analysis

Data analysis and result mapping were conducted using IBM SPSS Statistics for Windows, version 27 (IBM Corp., Armonk, N.Y., USA) and Origin 2021. Factors potentially affecting patient prognosis were analyzed using multivariate Cox regression, and a forest plot was generated to illustrate the results. The cumulative incidence of liver disease progression across groups was plotted using Kaplan–Meier curves, and differences were compared using the log-rank test. Differences in MVR between groups were evaluated using the chi-square test. Statistical significance was set at *p* < 0.05.

## 3 Results

### 3.1 Study population

**FIGURE 1:**
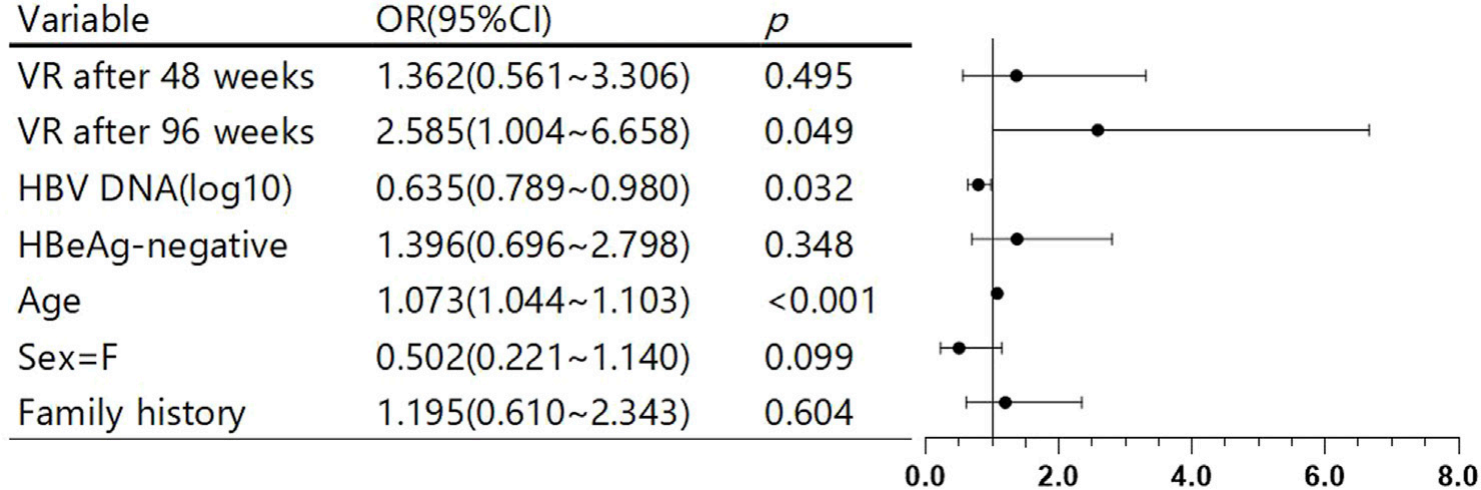
Multivariate Cox analysis of factors affecting patient prognosis and forest plot.

This study included 1,037 eligible patients with CHB, with a retrospective follow-up period of 17.3 years. Among them, 847, 138, and 52 patients were in the within-48-week, 48–96-week, and after-96-week groups, respectively. The median follow-up durations for these groups were 6.32 (4.95) years, 6.31 (5.29) years, and 6.26 (6.20) years, respectively, with no statistically significant differences after pairwise comparison. Additionally, the mean age of the patients was 35.7 (75% confidence interval, 29–41) years. The cohort included 762 male and 275 female patients. Among them, 669 and 368 received ETV antiviral and tenofovir treatments, respectively. At 48 weeks, 84.1% and 76.5% of patients receiving ETV and tenofovir, respectively, achieved VR. Overall, 81.6% of the study population achieved VR within 48 weeks; 522 and 515 had HVL and no HVL, respectively. Initially, 668 patients had HBeAg-positive status, and 369 had HBeAg-negative status. After conducting a multivariate Cox analysis of factors that may affect patient prognosis (Figure 1)—including patient’s VR time, baseline viral load, HBeAg status, gender, age, and family history—we found that compared with patients who achieved VR within 48 weeks, those who achieved VR between 48 and 96 weeks did not have a significantly increased risk of poor prognosis. However, VR after 96 weeks was a risk factor for poor prognosis compared with VR within 48 weeks (OR = 2.585, p = 0.049). Additionally, increasing age (OR = 1.073, p < 0.001) was a risk factor for poor prognosis, while higher viral load was a protective factor (OR = 1.635, p = 0.032). No other factors showed statistically significant associations.

### 3.2 Total cohort

**FIGURE 2:**
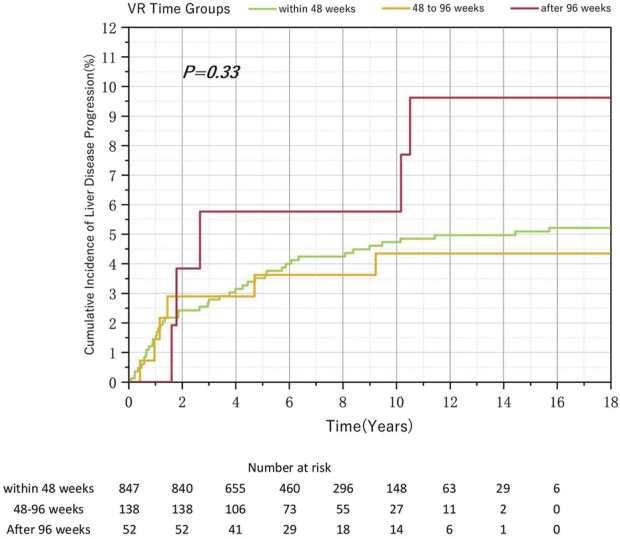
Relationship between the virological response time to Nucleos(t)ide analog therapy and the cumulative incidence of liver disease progression in chronic hepatitis B.

Among the 1,037 patients with CHB enrolled in this study, 847 (81.6%), 138 (13.3%), and 52 (5.0%) achieved VR within 48, 48–96, and after 96 weeks, respectively. During the follow-up period, liver disease progression was observed in 43 (5.1%), 6 (4.4%), and 5 (9.6%) patients in the within-48-week, 48–96-week, and after-96-week groups, respectively. Although the cumulative incidence rate of liver disease progression was highest in the after-96-week group, no significant differences were observed (*p* = 0.33) (Figure 2). Pairwise comparisons revealed no significant differences. However, the after-96-week group exhibited the highest rate of MVR incidence, with a significant difference (*p* < 0.05) (Table 1). The incidence of liver disease progression among the 125 patients who failed to achieve MVR (8%) and that of patients who achieved MVR (4.82%) did not reach statistical significance (*p* = 0.134) (Supplementary Table S1).

**TABLE 1:** Relationship between the virological response time to Nucleos(t)ide analog therapy and the rate of MVR in chronic hepatitis B.

*Group*	*Total*	*MVR(n,%)*	*Non-MVR(n,%)*	*Chi-square*	*p-value*
*Within-48-week*	*847*	*744(87.8%)*	*103(12.2%)*	*6.346*	*0.042*
*48–96-week*	*138*	*117(84.8%)*	*21(15.2%)*		
*After-96-week*	*52*	*51(98.1%)*	*1(1.9%)*		

MVR: maintained virological response.

### 3.3 Patients with HVL at baseline

**FIGURE 3:**
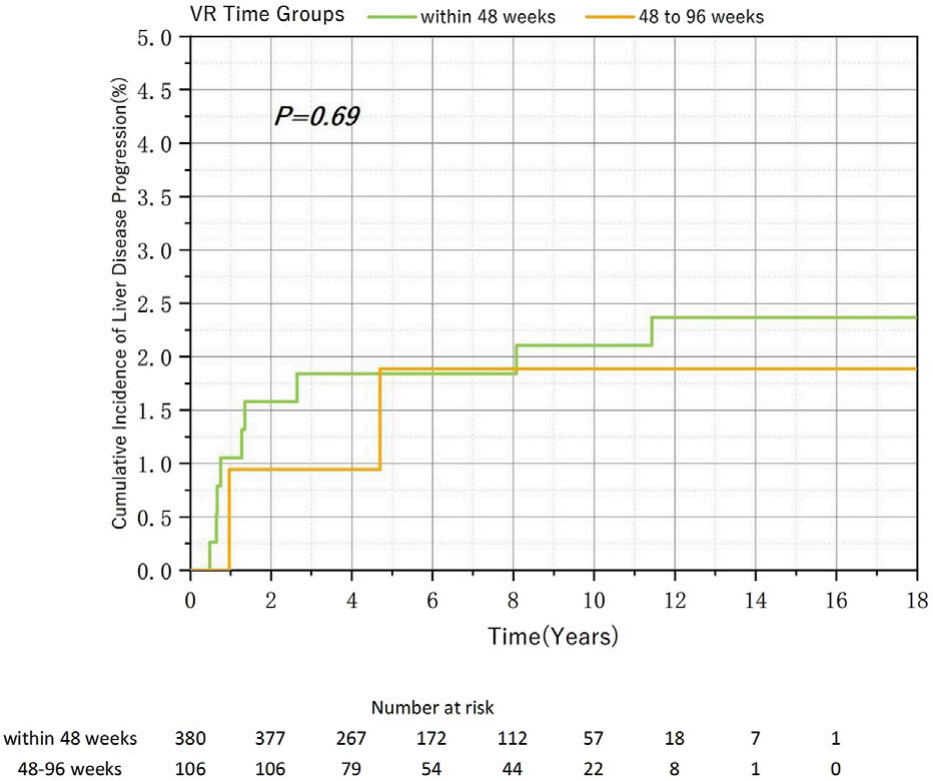
Relationship between the virological response time to Nucleos(t)ide analog therapy and the cumulative incidence of liver disease progression in chronic hepatitis B and baseline high viral load.

Overall, 522 patients with CHB and HVL at baseline were included in this study. Among them, 380 (72.8%), 106 (20.3%), and 36 (6.9%) patients achieved VR within 48, 48–96, and after 96 weeks, respectively. Owing to the small sample size in the after-96-week group and previous findings showing no significant differences, we compared the cumulative incidence of liver disease progression (Figure 3) and MVR (Table 2) rates between the within-48-week and 48–96-week groups. No significant association was found between these outcomes and the time required to achieve VR.

**TABLE 2:** Relationship between the virological response time to Nucleos(t)ide analog therapy and the rate of MVR in chronic hepatitis B and baseline high viral load.

*Group*	*Total*	*MVR(n,%)*	*Non-MVR(n,%)*	*Chi-square*	*p-value*
*Within-48-week*	*380*	*332(87.4%)*	*48(12.6%)*	*0.025*	*0.875*
*48–96-week*	*106*	*92(86.8%)*	*14(13.2%)*		

MVR: maintained virological response.

### 3.4 Patients without HVL at baseline

**FIGURE 4:**
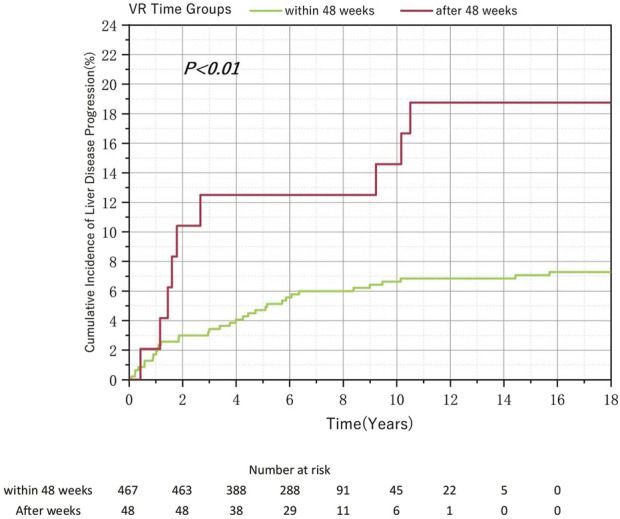
Relationship Between the Virological Response Time to Nucleos(t)ide Analog Therapy and the Cumulative Incidence of Liver Disease Progression in Chronic Hepatitis B and Baseline non-High Viral Load.

Of the 515 patients without HVL at baseline enrolled in this study, 467 (90.7%) achieved VR within 48 weeks. Given that over 90% of patients achieved VR within 48 weeks, we merged the 48–96 weeks and the after-96-week groups into a single “after-48-week” group. The analysis revealed that the cumulative incidence of liver disease progression rate was significantly higher in the after-48-week group than in the within-48-week group. Despite the significant differences in sample size, the log-rank test confirmed a statistically significant difference between the two groups (Figure 4). However, the difference in the MVR rates was not statistically significant (Table 3).

**TABLE 3:** Relationship Between the Virological Response Time to Nucleos(t)ide Analog Therapy and the Rate of MVR in Chronic Hepatitis B and Baseline non-High Viral Load.

*Group*	*Total*	*MVR(n,%)*	*Non-MVR(n,%)*	*Chi-square*	*p-value*
*Within-48-week*	*467*	*412(88.2%)*	*55(11.8%)*	*0.324*	*0.640*
*After-48-week*	*48*	*41(85.4%)*	*7(14.6%)*		

MVR: maintained virological response.

### 3.5 Patients with baseline HBeAg-Positive status

**FIGURE 5:**
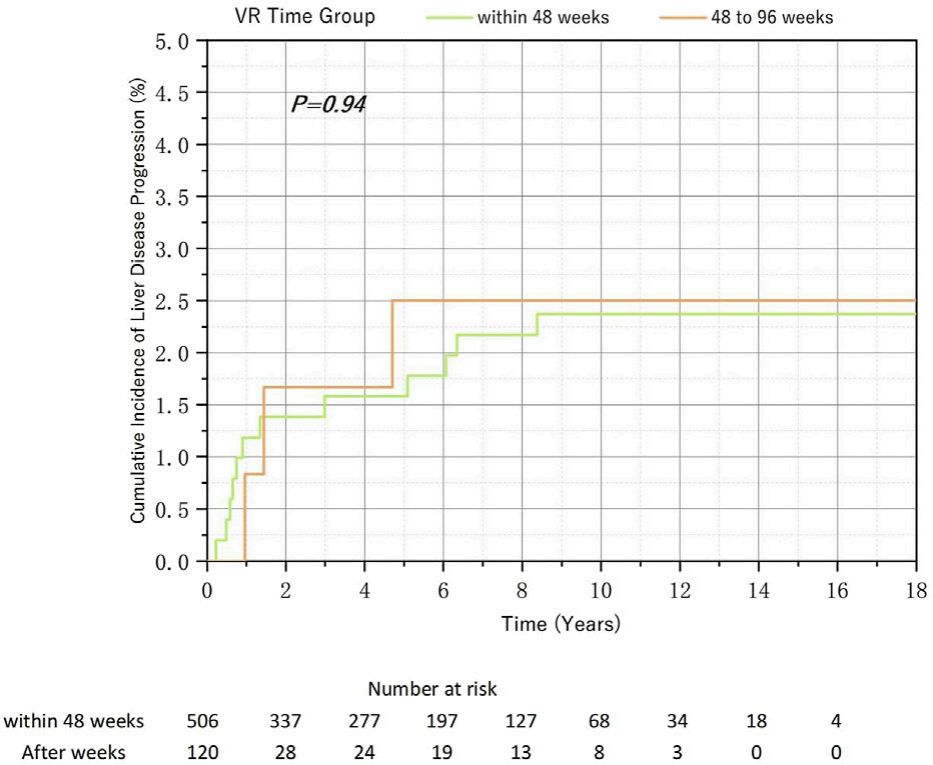
Relationship between the virological response time to Nucleos(t)ide analog therapy and the cumulative incidence of liver disease progression in chronic hepatitis B and baseline HBeAg-positive.

Overall, 668 patients with positive HBeAg at baseline were included. Among them, 506 patients (75.7%) achieved VR within 48 weeks, 120 patients (18.0%) achieved VR between 48 and 96 weeks, and 42 patients (6.3%) achieved VR after 96 weeks. Since the number of patients with a response time exceeding 96 weeks was small and previous studies had confirmed that the cumulative incidence of disease progression in the after-96-week group in the total population was higher, but the difference was not significant, we only analyzed the within-48-week and between-48–96-week groups. The cumulative incidences of liver disease progression in the two groups were 2.37% and 3.09%, respectively. This difference, as shown on the Kaplan-Meier (K-M) curve, was not statistically significant (Figure 5). Additionally, there was no statistically significant difference in the MVR rate among the groups (Table 4).

**TABLE 4:** Relationship between the virological response time to Nucleos(t)ide analog therapy and the rate of MVR in chronic hepatitis B and baseline HBeAg-positive.

*Group*	*Total*	*MVR(n,%)*	*Non-MVR(n,%)*	*Chi-square*	*p-value*
*Within-48-week*	*506*	*444(87.75%)*	*62(12.25%)*	*0.322*	*0.570*
*48–96-week*	*120*	*103(85.83%)*	*17(14.17%)*		

MVR: maintained virological response.

### 3.6 Patients with baseline HBeAg-Negative status

**FIGURE 6:**
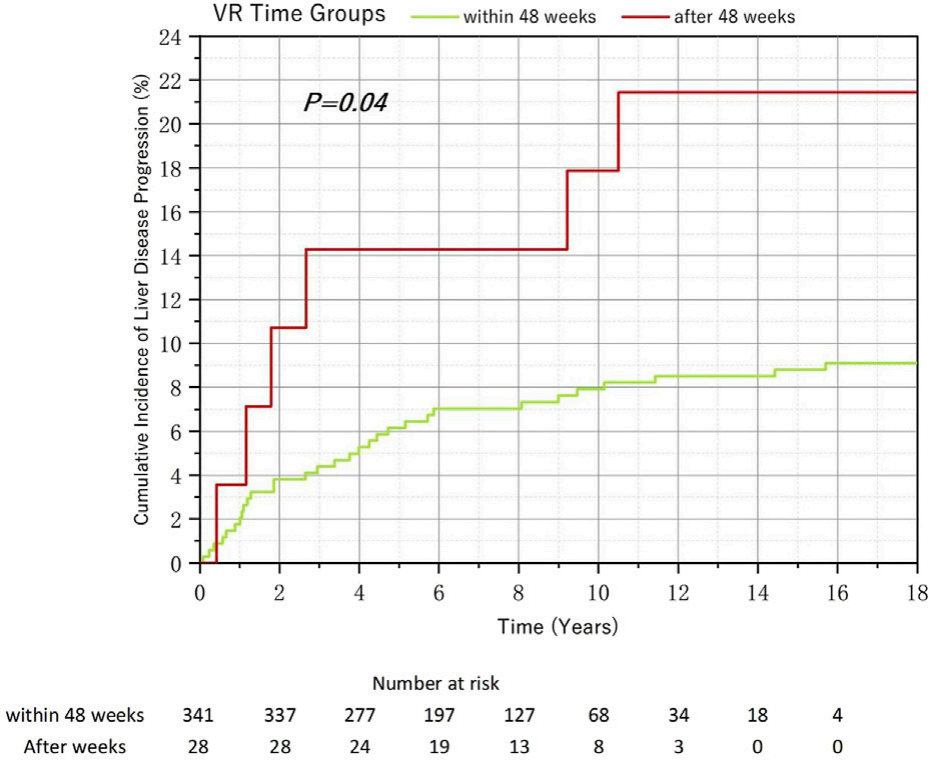
Relationship between the virological response time to Nucleos(t)ide analog therapy and the cumulative incidence of liver disease progression in chronic hepatitis B and baseline HBeAg-negative.

Among HBeAg-negative patients, 341 individuals (92.41%) achieved VR within 48 weeks. For reasons similar to those in the non-HVL investigation, we combined the 48–96-week group and the after-96-week group. Despite the disparity in numbers, the subsequent risk of liver disease progression events was even more pronounced, with 9.09% of the within-48-week group patients and 21.43% of the after-48-week group patients experiencing disease progression. This difference was statistically significant according to the Kaplan-Meier (K-M) curve analysis (Figure 6; *p* < 0.05). Regarding the analysis of the incidence of MVR, there was no significant difference between groups (Table 5).

**TABLE 5:** Relationship between the virological response time to Nucleos(t)ide analog therapy and the rate of MVR in chronic hepatitis B and baseline HBeAg-negative.

*Group*	*Total*	*MVR(n,%)*	*Non-MVR(n,%)*	*Chi-square*	*p-value*
*Within-48-week*	*341*	*300(87.98%)*	*41(12.02%)*	*0.124*	*0.762*
*After-48-week*	*28*	*24(85.71%)*	*4(14.29%)*		

MVR: maintained virological response.

## 4 Discussion

Currently, NAs are the frontline antiviral therapy for hepatitis B and exhibit remarkable efficacy. Over 90% of patients achieve VR within 96 weeks of monotherapy while significantly reducing the risk of disease progression (European Association for the Study of the Liver and EASL, 2017; Terrault et al., 2018; Lau et al., 2021; You et al., 2023). Research on VR timing primarily focuses on factors such as baseline viral levels and HBeAg status (Marcellin et al., 2008; Fung et al., 2015; Hou et al., 2015; Yan et al., 2015; Byrne et al., 2018; Liu et al., 2021). Although the reported VR rates within 48 weeks vary across studies, patients with HVL consistently exhibit lower VR rates than those without HVL (Fung et al., 2015; Yan et al., 2015). Our study’s results support these findings. The rates of achieving VR within 48 weeks were 72.5% and 90.7% for patients with HVL and those without HVL at baseline, respectively. The rates of achieving VR within 48 weeks were 75.7% and 92.4% for HBeAg-positive and HBeAg-negative patients at baseline, respectively. Notably, 71.5% of patients with prolonged VR times had HVL and HBeAg-positive status. When the observation period was extended to 96 weeks, the VR rates in all groups were increased to over 90%.

Patients who achieved VR after 96 weeks demonstrated a higher cumulative incidence of liver disease progression than those who achieved VR within 96 weeks. However, this difference was not statistically significant because of the small sample size, suggesting that a larger sample size might show meaningful changes. Among HVL or HBeAg-positive patients, no statistically significant difference was observed in the cumulative incidence of liver disease progression between those who achieved VR within 48 and 48–96 weeks. Conversely, among non-HVL or HBeAg-negative patients, despite a significant difference in sample size, the cumulative incidence of liver disease progression was significantly higher in the after-48-week group than in the within-48-week group. In clinical practice, patients who do not achieve VR within an extended period and show signs of disease progression may undergo adjustments in their antiviral treatment regimens, potentially leading to their exclusion from our study. Therefore, the actual differences between groups may be even more pronounced.

Some patients may concurrently exhibit the characteristics of “HVL and HBeAg-negative” or “non-HVL and HBeAg-positive,” leading to uncertain outcomes. There were only 71 patients with “HVL and HBeAg-negative” status, and the sample size was too small for statistical analysis. A total of 217 patients had “non-HVL and HBeAg-positive” status, including 190 (87.56%) who achieved VR within 48 weeks, 19 (8.76%) who achieved VR between 48 and 96 weeks, and 8 (3.69%) who achieved VR after 96 weeks. Among them, 8 (4.21%), 1 (5.26%), and two patients (25%) experienced disease progression, respectively. When pooling patients who achieved VR after 48 weeks, there was no statistically significant difference in the incidence of liver disease progression between those who achieved VR within 48 weeks and those who achieved VR after 48 weeks (8/190, 4.2% vs 3/27, 11.11%; p = 0.126). The log-rank test for survival analysis also showed no statistical significance (p = 0.120) (Supplementary Figure S1). For clinical prudence, we recommend that patients who have either “non-HVL” or “HBeAg-negative” status aim for VR within 48 weeks for antiviral therapy.

When comparing MVR rates, patients who required >96 weeks to achieve VR appeared to have a higher likelihood of MVR. However, the MVR rates among the other groups, regardless of baseline viral load or HBeAg status, remained similar, ranging from 85% to 90%. This result can be attributed to several factors. First, the longer time to achieve VR reduced the observation period for potential virological breakthroughs after VR. Specifically, the mean follow-up after VR was significantly lower (3.67 (6.25) years) in patients who achieved VR after 96 weeks than in those who achieved VR within 96 weeks (5.98 (4.99), *p* < 0.01). Second, excluding cases in which drug changes occurred before VR was achieved might have introduced some bias. Whether MVR is achieved can significantly impact patient outcomes, as extensively discussed in previous studies (Jang et al., 2018; Nam et al., 2018; Zhang et al., 2021). In this study, the progression rate was higher in the non-MVR group, but the difference did not reach statistical significance. This may be related to the large variance in follow-up time after VR and the insufficient sample size. If the analysis were limited to patients with follow-up times between 240 and 480 weeks after VR, a statistically significant difference would emerge (20/406, 4.93% vs 6/37, 16.22%, *p* = 0.01). However, this approach would further exacerbate the issue of insufficient sample size.

We also found that the baseline viral load significantly affected the time required for patients to achieve VR and their overall prognosis. Patients with untreated HBeAg-negative status usually experience longer infection durations and worse prognoses than those with HBeAg-positive status (Hadziyannis and Papatheodoridis, 2006; Lau et al., 2021; You et al., 2023), with approximately 80% of these patients falling into the non-HVL group. A January 2023 study by the American Association for the Study of Liver Diseases, which included 7,545 patients, found that patients with HBeAg-positive status and HVL at treatment initiation had a lower subsequent hepatocellular carcinoma incidence rate than the other groups (Choi et al., 2024). This finding is currently attributed to the patient’s immune status: as the disease progresses, immune activation damages infected hepatocytes, which lowers HBV DNA levels but increases the likelihood of disease progression (Mason et al., 2010; Tu et al., 2021). In our study, patients with HVL at baseline had a significantly lower cumulative incidence of disease progression than those without HVL at baseline (2.11% vs 8.34%, *p* < 0.01). This suggests that extending the antiviral target time to achieve VR is relatively safe for patients with HVL, particularly those with HVL and HBeAg-positive status.

However, the small sample size limits our prognostic assessment of patients with VR beyond 96 weeks, and further research is needed.

In conclusion, our study underscores the importance of maintaining a 48-week VR target in patients without HVL. For patients with both HVL and HBeAg-positive status, we recommend extending the antiviral monotherapy duration to 96 weeks. This recommendation is supported by their relatively lower VR rates at 48 weeks, the lack of significant differences in disease progression or MVR achievement rates between the patients categorized by different VR timeframes, and their generally favorable prognosis, which is characterized by a low incidence of disease progression.

## Data Availability

The original contributions presented in the study are included in the article/Supplementary Material, further inquiries can be directed to the corresponding author.
