# Supplementary figures and images for: Relationship Between Nucleos(t)ide analogue antiviral response time and prognosis in Chronic Hepatitis B: conclusions depend on baseline viral load and HBeAg status
Source: Front Pharmacol. 2025 Apr 24;16:1572827. doi: 10.3389/fphar.2025.1572827 (PMC12058476; doi:10.3389/fphar.2025.1572827)

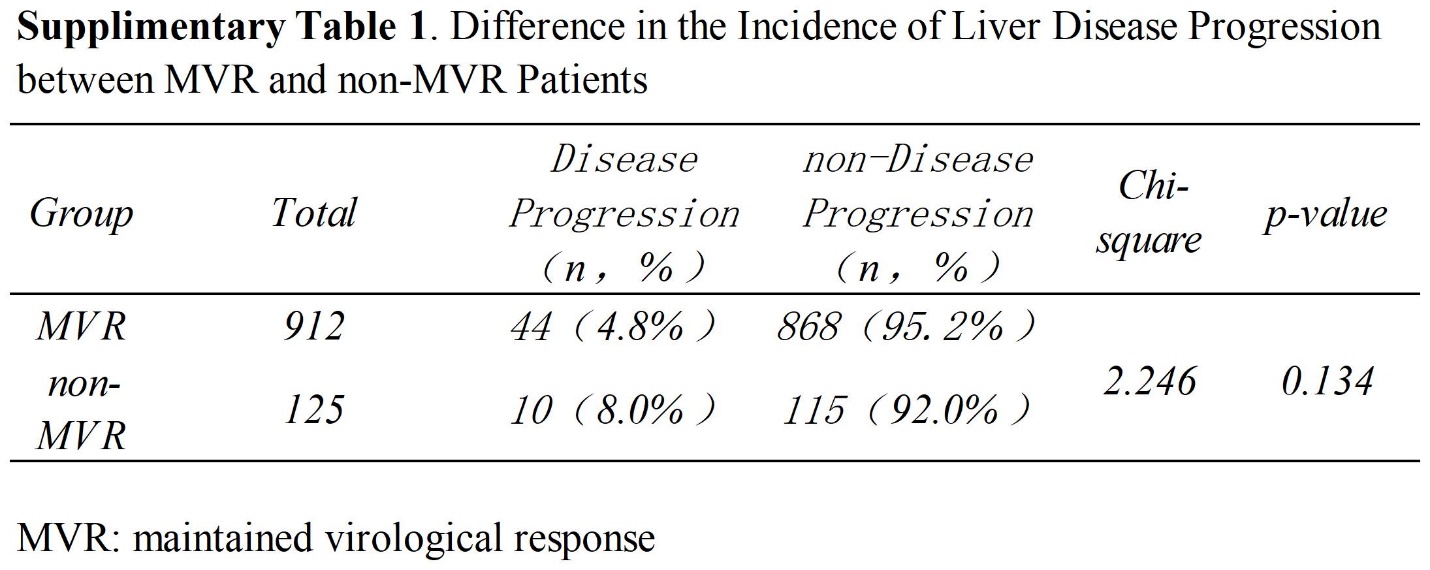

Supplement: Supplementary file 1 [file Table1.docx]

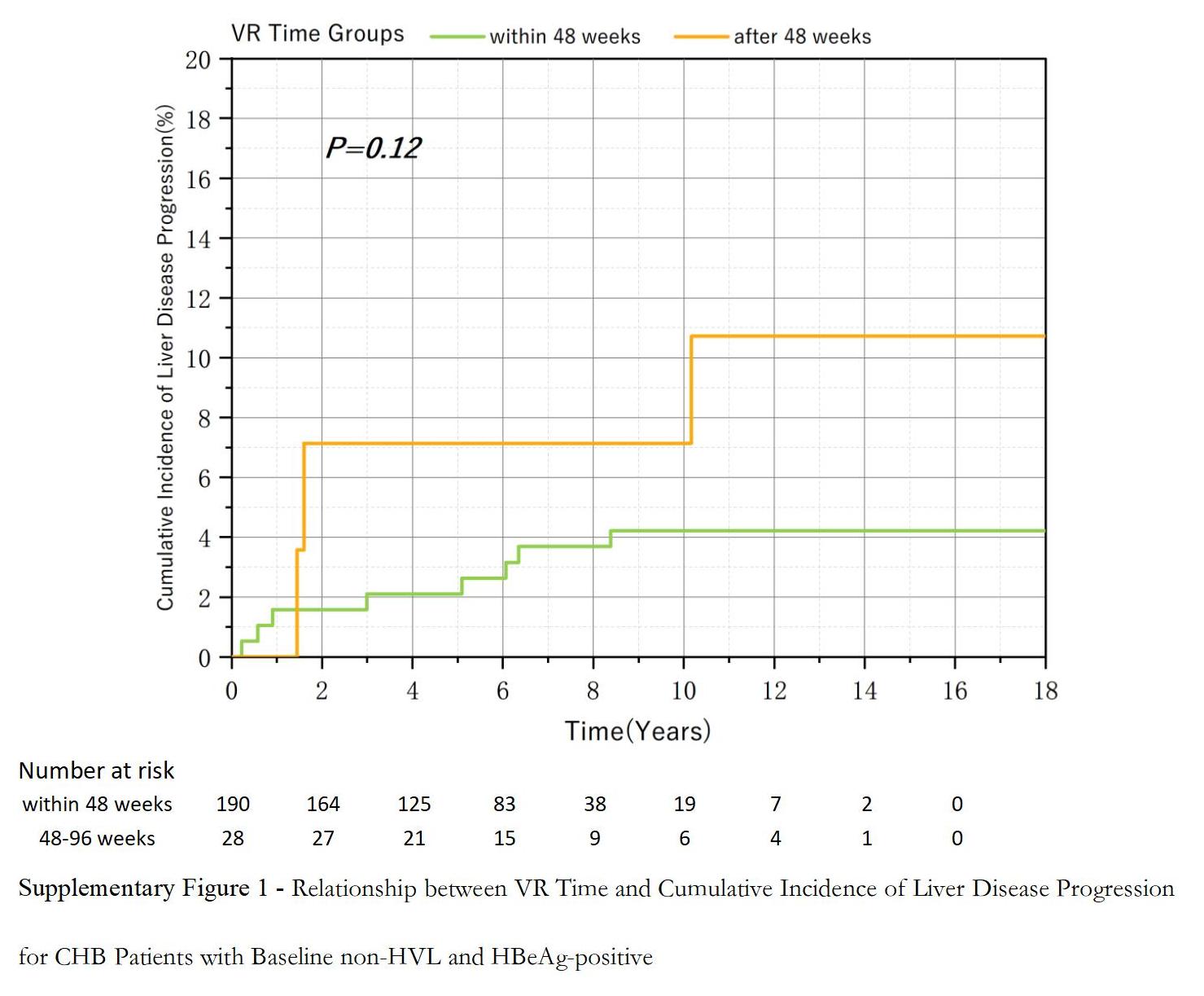

Supplement: Supplementary file 2 [file Image1.jpeg]
